# Hypertension care cascade in an urban resettlement colony and slum in Delhi, India: a cross-sectional survey

**DOI:** 10.1186/s12889-023-17021-8

**Published:** 2023-10-27

**Authors:** Mongjam Meghachandra Singh, Saurav Basu, Heena Lalwani, Shivani Rao, Vansh Maheshwari, Sandeep Garg, Nandini Sharma

**Affiliations:** 1 Department of Community Medicine, Maulana Azad Medical College, New Delhi, India; 2 Indian Institute of Public Health - Delhi, Public Health Foundation of India, New Delhi, India; 3 Department of Internal Medicine, Maulana Azad Medical College, New Delhi, India

**Keywords:** Hypertension, Adherence, Control, Care cascade, Screening, India

## Abstract

**Background:**

Hypertension care cascade in resource-limited settings is compromised with a majority of patients with hypertension remaining undiagnosed, untreated, non-adherent, and poorly controlled at every stage. However, there is paucity of information on care and management of hypertensive patients in community-based settings of low-income urban neighbourhoods in India.

**Methods:**

This was a community-based cross-sectional study conducted in an urban resettlement colony and slum area in the Northeast District of Delhi. The adult population was screened for hypertension using standardized methods, and adherence to medications was assessed using the Morisky Green Levine scale. Binary logistic regression analysis was conducted to ascertain the sociodemographic predictors of the outcome (presence of hypertension, adherence to antihypertensive medication, blood pressure control). A p-value < 0.05 was considered statistically significant.

**Results:**

We included 8850 adult participants including 5295 females and 3555 males in this study. Nearly 29% of the participants were hypertensive, of which 61.77% were newly diagnosed cases. Furthermore, nearly 81% of the previously diagnosed cases had been initiated on antihypertensive medication, of which 57.54% were adherent to their medications while 36.12% attained controlled blood pressure levels. The odds of having hypertension were significantly higher among males (AOR = 1.87, 95% CI: 1.63 to 2.15), age ≥ 60 years (AOR = 9.15, 95% CI: 7.82 to 10.70), high waist circumference (AOR = 2.24, 95% CI: 1.86 to 2.70) and Body Mass Index of ≥ 25.00 (AOR = 2.55, 95% CI: 2.00 to 3.26). Furthermore, on adjusted analysis, patients of hypertension having diabetes (DM) comorbidity had significantly higher odds of being adherent to anti-hypertensive medications (AOR = 1.81, 95% CI: 1.31 to 2.51) compared to those without DM comorbidity, while tobacco users had significantly lower odds of being adherent to antihypertensive medication (AOR = 0.50, 95% CI: 0.31 to 0.82).

**Conclusions:**

Hypertension care cascade in urban slum-resettlement colony setting revealed a high burden of undiagnosed hypertension, low rates of medication adherence, and poor blood pressure control. Strengthening community screening and primary care continuum of care is necessary to improve the hypertension care cascade from early diagnosis to effective management with optimal health outcomes to reduce patient complications and increase longevity.

## Background

Hypertension (HTN) signifying elevated blood pressure is an established risk factor for cardiovascular diseases and renal dysfunction resulting in premature and preventable mortality [1, 2]. Globally, more than one billion adults have hypertension with majority of them residing in low- and middle- income countries (LMICs) [3]. In India, evidence from a nationally representative survey indicate that at least one in four adults have hypertension [4]. Furthermore, there exists considerable regional variation in the prevalence of hypertension in India with nearly one-third of urban adults and one-fourth of rural adults having hypertension [5]. High burden of hypertension in India is attributed to the ongoing demographic transition and associated increasing aging population, and epidemiological transition related risk factors such as overweight/obesity, sedentary lifestyles, alcohol and tobacco consumption and comorbidities as some of the risk factors associated with hypertension [6].

To meet the sustainable objective goals for a one-third reduction in premature mortality from NCDs by 2030, the global NCD action plan target of a 25% relative reduction in the prevalence of high blood pressure in adults by 2025 is required [7, 8]. Hypertension screening, diagnosis, and effective pharmacological and lifestyle management to lower blood pressure is highly cost-effective and essential for prevention of major microvascular and macrovascular complications resulting from uncontrolled hypertension but the asymptomatic condition is frequently neglected by patients and health systems in the developing world [1, 2, 9]. Consequently, worldwide, there is a high prevalence of deficient hypertension management practices with nearly half the patients with hypertension remaining undiagnosed and not initiated on therapy, while merely one in five achieve optimal blood pressure control [10]. Evidence from studies evaluating the hypertension care cascades in LMICs signifying the extent of retention of hypertensive patients from the stages of screening, diagnosis, initiation of treatment, and attainment of blood pressure control indicate major losses at all stages representing an ongoing public health challenge [11–13]. Within LMICs, the pooled estimate of medication non-adherence to hypertension treatment was estimated as 47.34% after pooling data from 42 studies [14].

Within India, evidence from the fifth series of the National Family Health Survey (NFHS-5, 2019-21), a nationally representative survey in the 15–49 age-group, concluded that only 4 in 10 patients diagnosed with hypertension in India were initiated on anti-hypertensive treatment, while only 3 in 4 on treatment attained optimal blood pressure control [15]. In the first Indian national NCD survey (2018–19), the prevalence of hypertension in the 18–49 age-group was 28.5% of whom only 14.5% were receiving treatment, and just 12.6% had controlled blood pressure levels [16]. Among the elderly in India, evidence from the Longitudinal Study of Ageing observed that only one in two patients were initiated on treatment while blood pressure control was achieved in only one in four patients with hypertension [17]. As per a systematic review of evidence, only 22.5% of patients with hypertension in India have their blood pressure control [18]. A key driver for poorly controlled hypertension in LMICs including India is either non-initiation, delayed initiation, and among the initiated, non-adherence to antihypertensive medication due to multiple patient, provider, and health system factors [19, 20]. Consequently, it is estimated that improved hypertension control can prevent nearly 5,00,000 premature deaths annually in India [21].

Slums are areas of substandard housing and squalor which as per the WHO usually lacks basic amenities such as improved water and sanitation, with insufficiency of living area, non-durable housing, and no secure tenure, with nearly 130 million people in South Asia, including 49% of the total urban population in India living in slums due to unplanned urbanization and large scale rural to urban migration [22–24]. Urban resettlement colonies were created in mega-cities like Delhi to house people who were evicted during removal of slums, and are mostly overcrowded, lack adequate sanitation and hygiene [25]. It is estimated that Delhi includes nearly 34% people living in urban slums and 12% people living in resettlement colonies [26]. People living in urban slums and disadvantaged neighbourhoods experience multiple adverse determinants of health and risk factors for the development of hypertension high levels of stress, poor nutrition secondary to poverty translating to high salt diets with very low consumption of fresh fruit and vegetables [27]. Studies in slum settings worldwide have reported disproportionately high prevalence of hypertension compared to the general population [28–33]. A study from Kolkata in India reported 42% prevalence of hypertension in an urban slum setting [30]. Furthermore, difficulties in accessibility, affordability, and availability of healthcare services in vulnerable slum populations may delay the screening and diagnosis of hypertension and maintaining adherence to treatment which accentuate the risk of uncontrolled hypertension and resultant complications [28–30].

In India, extensive screening of hypertension among adults through opportunistic screening in health facilities and community-based screening through frontline workers has been recommended by the National Programme for Prevention & Control of Non-Communicable Diseases (NPNCD) [31]. Although evidence from national surveys indicates deficient hypertension care and control cascade in India, there is paucity of disaggregated data from populations living in urban slums and resettlement colonies. Moreover, these studies do not collect information on antihypertensive medication adherence and their predictors [13, 15].

The objective of this study was to assess the hypertension care cascade and their predictors in populations living in urban slums and resettlement colonies in Delhi i.e., proportion of population with hypertension, proportion of hypertension patients initiated on antihypertensive treatment, proportion of hypertensive patients initiated on treatment that were adherent to treatment, and the proportion of patients on treatment that had controlled blood pressure levels.

## Methods

Design and Setting: This was a community-based cross-sectional study conducted in an urban resettlement colony and slum area in the Northeast District of Delhi having an estimated ~ 54,614 total population, a site purposively selected, as it represents the field practice area of a government medical college. The study area included Gokalpuri urban resettlement colony (~ 16,878), Sanjay Colony urban slum (~ 4467), Gokalpuri village (~ 8608), and the adjoining Ganga Vihar urban resettlement colony (~ 24,661). A Demographic Developmental and Environmental Surveillance Site (DDESS) was recently established in in the area inclusive of complete Geographic Information System (GIS) based mapping of the sociodemographic correlates of the study population. The household level response rate of the current survey was ~ 97% due to high levels of pre-existing community engagement [32].

Study Population: This study included all individuals aged ≥ 18 years who were residents of the area for at least 6 months irrespective of their medical history. Data were collected for a period of 2 months from March-April 2023.

Primary Outcome of the study was the detection of Hypertension (including both newly diagnosed or previously diagnosed cases). Hypertension was defined on screening as either a systolic blood pressure (SBP) ≥ 140 mm Hg or diastolic blood pressure (DBP) ≥ 90 mm Hg, or any individual who self-reported themselves as previously diagnosed patients of hypertension.

Secondary outcomes were the proportion of hypertensive patients initiated on treatment, proportion of patients initiated on antihypertensive medications that were adherent to their prescribed treatment, and the proportion who had controlled blood pressure values.

Operational definitions: Those participants who self-reported having hypertension diagnosed by any healthcare provider or currently taking any antihypertensive medication were recorded as ‘previously diagnosed hypertensive’. Newly diagnosed hypertensives were those detected with either SBP ≥ 140 or DBP ≥ 90 upon screening without past history or diagnosis of hypertension. Controlled blood pressure was considered as previously diagnosed hypertensive patients with SBP and DBP less than 140 and 90 mm of Hg, respectively [33].

Adherence to antihypertensive medications was assessed using the previously validated four-item Morisky Green Levine (MGL) adherence scale [34]. The MGL scale comprises four questions (pertaining to forgetfulness or carelessness, cessation of prescribed medications when feeling better or worse) where each item has a yes or no response. In our study, we dichotomized the full score on the MGL scale into two groups; those who scored 4 on the MGL scale were considered as adherent and those with scores of < 4 was considered as non-adherent.

Sample Size and Sampling Strategy: The sample size was adequate at 95% confidence levels, 3% absolute precision, design effect of 3.5 considering the heterogeneity in the slum and expecting 50% expected prevalence of adherence to antihypertensive medication [20]. The entire study area was divided into 16 sectors (clusters). The village and urban slum clusters were selected purposively to ensure survey representativeness, we did purposive (mandatory) selection of participants from the slum and village clusters due to expected heterogeneity in living standards and health behaviours between the slum, village, and resettlement colony population. Three additional clusters from the urban resettlement colony were selected through simple random sampling. House-to-house sampling in the households within the selected clusters was conducted and all eligible and available participants in every open household were recruited in the study. Households that were locked were visited for a second time after approximately a week and nearly 50 houses that were locked even on second visit were excluded.

Methodology: Face to face interviews with respondents in the households were conducted with the participants by a total of ten trained field investigators in the local language, Hindi, using a pretested structured questionnaire. Electronic data collection using EpiCollect android application was conducted which has features of both online and offline data collection and data validation with facility for real-time monitoring [35]. Weight of the participants was measured with digital weighing scales with least count 0.1 kg, while waist circumference and height were measured using a measuring tape with least count of 1 cm. All the investigators were trained to measure weight, height, and blood pressure in the field settings using standard guidelines to reduce chances of measurement errors.

Supervision of the field investigators and quality assurance with random verification in 5% of the households was conducted by the field supervisor and the project coordinator while data quality was maintained through regular assessment and feedback provided by the project data manager.

Blood pressure (BP) measurement: The BP in all participants was measured as per standard guidelines [36] by the trained field investigators using an Omron Blood Pressure monitor (OMRON, Kyoto, Japan). Three blood pressure measurements with five-minute intervals between readings at rest were recorded, and the average of the second and third readings was considered as the estimated blood pressure.

### Independent variables

The following independent variables were considered in the analysis based on association with the study outcomes observed in previous studies [1, 13–16, 29].

Age of the participants was stratified into young (18–39), middle (40–59), and older adults (≥ 60 years), gender (male/female): education (stratified into illiterate; those who had studied up to primary school; those who had studied up to secondary school, and those who had studied beyond high school). The income level of the respondents was categorized as those with a monthly household income of median and below (< Rs 46,089) and above median (> Rs 46,095) value of the study sample.

Those who reported currently using tobacco in any form (either smoking or smokeless) were categorized as tobacco users.

Alcohol intake was measured using a single close ended item “Do you consume Alcohol” with options, “Never consume”, “Consume daily”, and “Consume occasionally”. The participants reporting consumption of alcohol either daily or occasionally were classified as “Yes”, or else as “No”.

Participants with absence of regular exercise (at least brisk walking for at least 5 days and 30 min per day) or in occupations not involving vigorously intensive activities were considered as having sedentary lifestyle, while those involved in regular exercise or involved in occupations involving vigorously intensive activities were considered as not having a sedentary lifestyle.

Family history of Hypertension was categorized as Yes (if one or both parents were previously diagnosed as having hypertension) and No (if none of the parents of the participant had been diagnosed as having hypertension).

Body mass index (BMI) was categorized using the Pan Asian classification: underweight (BMI < 18.5), normal (BMI: 18.5–22.9), overweight (BMI: 23.0–24.9), and obese (BMI ≥ 25.0) [37].

Waist circumference was measured for each participant and categorized as Low (< 80 cm for females and < 94 cm for males), Moderate (80-87.9 cm for females and 94-101.9 cm for males) and High (≥ 88 cm for females and ≥ 102 cm for males) [38].

### Data and statistical analysis

The dataset was cleaned using MS-Excel 365. Descriptive statistics were performed to show the distribution of variables and provide summary statistics. Bivariate analysis was conducted by testing for association of the outcomes (hypertension present, hypertension control, adherence to antihypertensive medication) with the independent variables using chi-square for categorical and independent samples t-test for continuous variables. Furthermore, a binary logistic regression was performed to ascertain independent association with outcome variables. The variables which on test for association had a P-value of < 0.20 in the bivariate analysis were entered into the multivariable analysis. Since education levels have been found to be associated with hypertension in most previous studies, it was included into multivariate model despite having a P-value > 0.20 in the unadjusted analysis. Both unadjusted odds ratio and adjusted odds ratio (AOR) with 95% CI were reported. For the final model, 5% was considered as the statistical significance level. Additionally, a multivariable linear regression was performed to check for associations of sociodemographic and lifestyle variables with systolic and diastolic blood pressure readings of the participants, wherein unadjusted and adjusted B-coefficients with 95% CI were reported. Model assumptions for both regression analysis such as multicollinearity, outliers and goodness of fit of each model was checked. All analysis were performed using Stata (Version 15.0, StataCorp, TX, USA).

### Ethics

The study was approved by the Institutional Ethics Committee. All participants provided written and informed consent. Patients detected with Hypertension on screening with either suboptimal medication adherence or lack of initiation on treatment were briefly counselled on health risks of uncontrolled hypertension, and referred to their nearby local government health facilities for further evaluation and management.

## Results

The present study screened 8850 adult participants for hypertension including 5295 females and 3555 males. The household response rate of the survey was ~ 97%. Table 1 reports the distribution of the sociodemographic, lifestyle, and clinical characteristics of the participants. A majority of the participants were females (59.83%), aged 18–39 years (58.96%), and educated above secondary level (61.17%). A total of 563 (6.36%, 95% CI: 5.87 to 6.89) participants were previously diagnosed for diabetes mellitus (DM).

**Table 1:** Sociodemographic, lifestyle and clinical characteristics of the participants (N = 8850)

Variables	n (%)
**Sex**	
Male	3555 (40.17)
Female	5295 (59.83)
**Age (years)**	
18–39	5218 (58.96)
40–59	2514 (28.41)
≥ 60	1118 (12.63)
**Educational level**	
Illiterate	1912 (21.60)
Primary	1524 (17.22)
Secondary	2758 (31.16)
High school certificate and above	2656 (30.01)
**Per Capita Income**	
Median and Below (< Rs 46,089)	8260 (93.33)
Above Median (> Rs 46,095)	590 (6.67)
**Waist Circumference**	
Low (< 80 cm for females and < 94 cm for males)	4826 (54.53)
Moderate (80-87.9 cm for females and 94-101.9 cm for males)	1738 (19.64)
High (≥ 88 cm for females and ≥ 102 cm for males)	2286 (25.83)
**BMI**	
< 18.5	1027 (11.60)
18.5 < 23	2841 (32.11)
23 < 24.9	1471 (16.62)
≥ 25.0	3511 (39.67)
**Tobacco consumption**	
Yes	1285 (14.52)
No	7565 (85.48)
**Alcohol consumption**	
Yes	1089 (12.31)
No	7761 (87.69)
**Sedentary lifestyle**	
Yes	2171 (24.53)
No	6679 (75.47)
**Family history of HTN**	
Yes	1139 (12.87)
No	7711 (87.13)
**DM comorbidity**	
Yes	563 (6.36)
No	8287 (93.64)

**Fig. 1:**
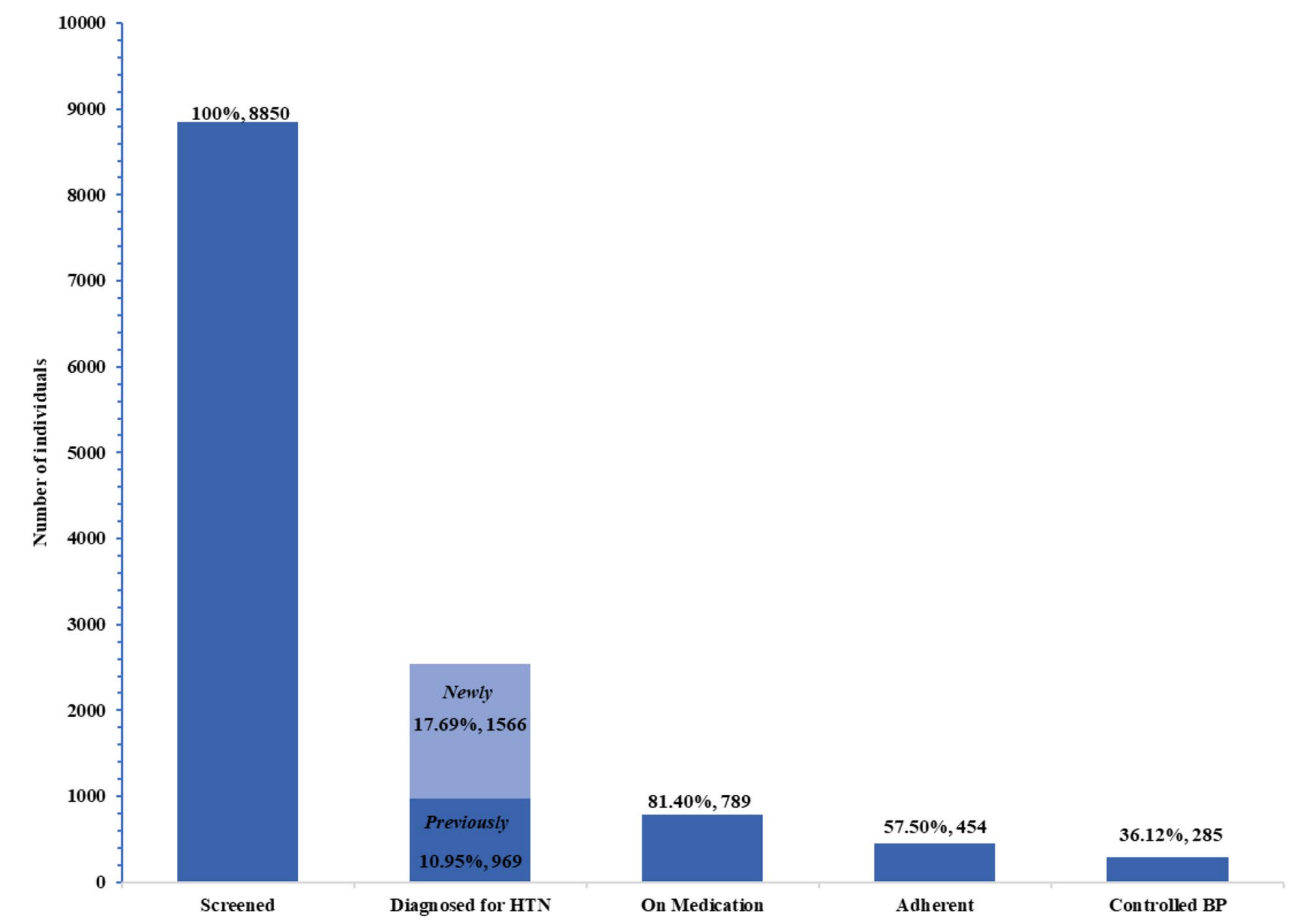
Hypertension care cascade in an urban slum-resettlement colony in Delhi, India

The prevalence of hypertension in the sample was 28.64% (n = 2535, 95% CI: 27.71 to 29.60) including 969 (10.95%) previously diagnosed and 1566 (17.69%) newly diagnosed cases (Fig. 1). More than two-third (68.11%) of the previously diagnosed cases were females and above 40 years of age (88.34%). A majority of the newly diagnosed cases were males (53.38%), and middle and elderly (57.85%) aged participants, and those having secondary education and above (60.22%) (Table 2). Model 1 reports the factors associated with new cases of hypertension (n = 1566) detected during screening in those without a prior diagnosis of hypertension. Male gender (AOR = 2.25, 95% CI: 1.93 to 2.63) and older age (AOR = 4.06, 95% CI: 3.36 to 4.92) were factors with statistically significant association with undiagnosed hypertension. Lifestyle characteristics such as high waist circumference (AOR = 1.90, 95% CI: 1.54 to 2.35), overweight/obesity (AOR = 2.72, 95% CI: 2.06 to 3.58) and alcohol consumption (AOR = 1.29, 95% CI: 1.05 to 1.57) were also positively associated with the cases of hypertension diagnosed on screening. Model 2 reports the factors associated with total cases of hypertension. On adjusted analysis, the odds of having hypertension were significantly higher among males (AOR = 1.87, 95% CI: 1.63 to 2.15) compared to females, and those aged ≥ 60 years (AOR = 9.15, 95% CI: 7.82 to 10.70) compared to those aged 18–39 years. Moreover, high waist circumference (AOR = 2.24, 95% CI: 1.86 to 2.70) and BMI of ≥ 25.0 (AOR = 2.55, 95% CI: 2.00 to 3.26) was associated with significantly higher odds of having hypertension. The odds of hypertension were also 1.3 times higher among alcohol consumers (AOR = 1.26, 95% CI: 1.04 to 1.51) compared to those reporting not consuming alcohol.

**Table 2:** Distribution of factors associated with prevalence of HTN (N = 8850)

Variables	HTN present				Model 1: Newly diagnosed cases versus no HTN		Model 2: Total HTN cases versus no HTN	
	No (n = 6315) n (%)	Previously diagnosed (old) cases (n1 = 969) n (%)	Newly diagnosed (n = 1566) n (%)	Total (both new and old cases) (n = 2535) n (%)	Unadjusted OR (95% CI), P-Value	Adjusted^a^ OR (95% CI), P-Value	Unadjusted OR (95% CI), P-Value	Adjusted^a^ OR (95% CI), P-Value
**Sex**					**P < 0.001**	**P < 0.001**	**P < 0.001**	**P < 0.001**
Male	2410 (38.16)	309 (31.89)	836 (53.38)	1145 (45.17)	1.86 (1.66, 2.07)	2.25 (1.93, 2.63)	1.33 (1.22, 1.47)	1.88 (1.64, 2.16)
Female	3905 (61.84)	660 (68.11)	730 (46.62)	1390 (54.83)	Ref	Ref	Ref	Ref
**Age (years)**					**P < 0.001**	**P < 0.001**	**P < 0.001**	**P < 0.001**
18–39	4445 (70.39)	113 (11.66)	660 (42.15)	773 (30.49)	Ref	Ref	Ref	Ref
40–59	1449 (22.95)	432 (44.58)	633 (40.42)	1065 (42.01)	2.94 (2.60, 3.33)	2.33 (2.04, 2.66)	4.23 (3.79, 4.72)	3.34 (2.97, 3.75)
≥ 60	421 (6.67)	424 (43.76)	273 (17.43)	697 (27.5)	4.37 (3.67, 5.19)	4.06 (3.36, 4.92)	9.52 (8.25, 10.98)	9.07 (7.76, 10.61)
**Educational level**					P = 0.524		**P = 0.0358**	P = 0.6989
Illiterate	1315 (20.82)	253 (26.11)	344 (21.97)	597 (23.55)	Ref		Ref	Ref
Primary	1092 (17.29)	153 (15.79)	279 (17.82)	432 (17.04)	0.98 (0.82, 1.17)		0.87 (0.75, 1.01)	0.90 (0.77, 1.07)
Secondary	2003 (31.72)	286 (29.51)	469 (29.95)	755 (29.78)	0.90 (0.77, 1.04)		0.83 (0.73, 0.94)	0.94 (0.82, 1.09)
High school certificate and above	1905 (30.17)	277 (28.59)	474 (30.27)	751 (29.63)	0.95 (0.81, 1.11)		0.87 (0.76, 0.99)	0.95 (0.82, 1.10)
**Per Capita Income**					P = 0.374		P = 0.925	
Median and Below (< Rs 46,089)	5893 (93.32)	896 (92.47)	1471 (93.93)	2367 (93.37)	Ref		Ref	
Above Median (> Rs 46,095)	422 (6.68)	73 (7.53)	95 (6.07)	168 (6.63)	0.90 (0.72, 1.13)		0.99 (0.82, 1.19)	
**Waist Circumference**					**P < 0.001**	**P < 0.001**	**P < 0.001**	**P < 0.001**
Low (< 80 cm for females and < 94 cm for males)	3894 (61.66)	249 (25.7)	683 (43.61)	932 (36.77)	Ref	Ref	Ref	Ref
Moderate (80-87.9 cm for females and 94-101.9 cm for males)	1143 (18.1)	206 (21.26)	389 (24.84)	595 (23.47)	1.94 (1.69, 2.23)	1.58 (1.32, 1.90)	2.17 (1.92, 2.46)	1.62 (1.38, 1.91)
High (≥ 88 cm for females and ≥ 102 cm for males)	1278 (20.24)	514 (53.04)	494 (31.55)	1008 (39.76)	2.20 (1.93, 2.51)	1.90 (1.54, 2.35)	3.30 (2.95, 3.68)	2.34 (1.95, 2.81)
**BMI**					**P < 0.001**	**P < 0.001**	**P < 0.001**	**P < 0.001**
< 18.5	895 (14.17)	40 (4.13)	92 (5.87)	132 (5.21)	Ref	Ref	Ref	Ref
18.5 < 23	2252 (35.66)	212 (21.88)	377 (24.07)	589 (23.23)	1.63 (1.28, 2.07)	1.63 (1.27, 2.09)	1.77 (1.45, 2.17)	1.70 (1.36, 2.12)
23 < 24.9	1037 (16.42)	152 (15.68)	282 (18.01)	434 (17.12)	2.64 (2.06, 3.40)	2.24 (1.71, 2.94)	2.84 (2.29, 3.52)	2.17 (1.70, 2.76)
≥ 25.0	2131 (33.75)	565 (58.31)	815 (52.04)	1380 (54.44)	3.72 (2.96, 4.68)	2.72 (2.06, 3.58)	4.39 (3.61, 5.34)	2.55 (2.00, 3.26)
**Tobacco consumption**					**P < 0.001**	P = 0.188	**P < 0.001**	P = 0.9298
Yes	823 (13.03)	113 (11.66)	349 (22.29)	462 (18.22)	1.91 (1.66, 2.20)	1.14 (0.94, 1.38)	1.49 (1.31, 1.68)	1.01 (0.85, 1.20)
No	5492 (86.97)	856 (88.34)	1217 (77.71)	2073 (81.78)	Ref	Ref	Ref	Ref
**Alcohol consumption**					**P < 0.001**	**P = 0.013**	**P < 0.001**	**P = 0.017**
Yes	694 (10.99)	86 (8.88)	309 (19.73)	395 (15.58)	1.99 (1.72, 2.31)	1.29 (1.05, 1.57)	1.49 (1.31, 1.71)	1.25 (1.04, 1.51)
No	5621 (89.01)	883 (91.12)	1257 (80.27)	2140 (84.42)	Ref	Ref	Ref	Ref
**Sedentary lifestyle**					**P = 0.0109**	P = 0.799	**P < 0.001**	P = 0.707
Yes	1478 (23.4)	278 (28.69)	415 (26.5)	693 (27.34)	1.18 (1.04, 1.34)	0.98 (0.86, 1.13)	1.23 (1.11, 1.37)	1.02 (0.91, 1.15)
No	4837 (76.6)	691 (71.31)	1151 (73.5)	1842 (72.66)	Ref	Ref	Ref	Ref
**Family history of HTN**					P = 0.2111		P = 0.145	
Yes	792 (12.54)	132 (13.62)	215 (13.73)	347 (13.69)	1.11 (0.94, 1.30)		1.11 (0.97, 1.27)	
No	5523 (87.46)	837 (86.38)	1351 (86.27)	2188 (86.31)	Ref		Ref	

Model 2: Goodness of Fit, P = 0.1811

Model 2: Goodness of Fit, P = 0.064

^**a**^Adjusted for sex, age, education level, waist circumference, BMI, tobacco consumption, alcohol consumption and sedentary lifestyle

Among the previously diagnosed cases of hypertension (n = 969), 180 (18.58%, 95% CI: 16.25 to 21.15) patients were not on treatment i.e., not taking any antihypertensive medication to lower blood pressure, while among those taking antihypertensive medications (n = 789), only 454 (57.54%, 95% CI: 54.05 to 60.96) were adherent to their prescribed blood pressure lowering drugs. On adjusted analysis, patients of hypertension having DM comorbidity had significantly higher odds of being adherent to anti-hypertensive medications (AOR = 1.81, 95% CI: 1.31 to 2.51) compared to those without DM comorbidity, while tobacco users had significantly lower odds of being adherent to antihypertensive medication (AOR = 0.50, 95% CI: 0.31 to 0.82) (Table 3).

**Table 3:** Distribution of factors associated with adherence to HTN medication (N = 789)

Variables	Adherent to medication		Unadjusted OR (95% CI), P-Value	Adjusted^a^ OR (95% CI), P-Value
	No (n = 335) n (%)	Yes (n = 454) n (%)		
**Sex**			P = 0.823	
Male	111 (33.13)	147 (32.38)	Ref	
Female	224 (66.87)	307 (67.62)	0.97 (0.72, 1.30)	
**Age**			**P = 0.002**	P = 0.082
18–39	34 (10.15)	30 (6.61)	Ref	Ref
40–59	168 (50.15)	189 (41.63)	1.28 (0.75, 2.17)	1.14 (0.66, 1.97)
≥ 60	133 (39.7)	235 (51.76)	2.00 (1.17, 3.42)	1.58 (0.90, 2.77)
**Educational level**			P = 0.816	
Illiterate	93 (27.76)	118 (25.99)	Ref	
Primary	54 (16.12)	70 (15.42)	1.02 (0.65, 1.6)	
Secondary	88 (26.27)	133 (29.3)	1.19 (0.81, 1.75)	
High school certificate and above	100 (29.85)	133 (29.3)	1.05 (0.72, 1.53)	
**Per Capita Income**			P = 0.342	
Median and Below (< Rs 46,089)	311 (92.84)	429 (94.49)	Ref	
Above Median (> Rs 46,095)	24 (7.16)	25 (5.51)	0.76 (0.42, 1.35)	
**Waist Circumference**			P = 0.535	
Low (< 80 cm for females and < 94 cm for males)	88 (26.27)	104 (22.91)	Ref	
Moderate (80-87.9 cm for females and 94-101.9 cm for males)	66 (19.7)	97 (21.37)	1.24 (0.82, 1.9)	
High (≥ 88 cm for females and ≥ 102 cm for males)	181 (54.03)	253 (55.73)	1.18 (0.84, 1.67)	
**BMI**			P = 0.9459	
< 18.5	13 (3.88)	14 (3.08)	Ref	
18.5 < 23	66 (19.70)	90 (19.82)	1.27 (0.56, 2.87)	
23 < 24.9	54 (16.12)	73 (16.08)	1.26 (0.55, 2.89)	
≥ 25.0	202 (60.30)	277 (61.01)	1.27 (0.59, 2.77)	
**Tobacco consumption**			**P = 0.003**	**P = 0.006**
Yes	53 (15.82)	40 (8.81)	0.51 (0.33, 0.80)	0.50 (0.31, 0.82)
No	282 (84.18)	414 (91.19)	Ref	Ref
**Alcohol consumption**			P = 0.673	
Yes	34 (10.15)	42 (9.25)	0.90 (0.56, 1.45)	
No	301 (89.85)	412 (90.75)	Ref	
**Sedentary lifestyle**			**P = 0.001**	**P = 0.006**
Yes	83 (24.78)	163 (35.9)	1.70 (1.24, 2.33)	1.57 (1.14, 2.18)
No	252 (75.22)	291 (64.1)	Ref	Ref
**Family history of HTN**			P = 0.414	
Yes	48 (14.33)	56 (12.33)	0.84 (0.56, 1.27)	
No	287 (85.67)	398 (87.67)	Ref	
**DM comorbidity**			**P < 0.001**	**P < 0.001**
Yes	80 (23.88)	173 (38.11)	1.96 (1.43, 2.69)	1.81 (1.31, 2.51)
No	255 (76.12)	281 (61.89)	Ref	Ref

Model Goodness of Fit, P = 0.524

^**a**^ Adjusted for age, education level, tobacco consumption, sedentary lifestyle and DM comorbidity

Among previously diagnosed hypertensive patients taking antihypertensive medications (n = 789), only 285 (36.12%, 95% CI: 32.83, 39.54) patients achieved controlled blood pressure levels. On adjusted analysis, patients with higher BMI (AOR: 0.13, 95% CI: 0.05 to 0.34) had significantly lower odds of achieving blood pressure control. Furthermore, patients initiated and adhering to antihypertensive medications had nearly 1.5 times higher odds of attaining controlled blood pressure levels (AOR = 1.48, 95% CI: 1.08 to 2.02). Overall, 362 (37.36%, 95% CI: 34.36, 40.46) participants had controlled blood pressure among the previously diagnosed HTN cases (N = 969) (Table 4).

**Table 4:** Distribution of factors associated with control of blood pressure among those on HTN medication (N = 789)

Variables	Blood Pressure control (SBP < 140 and DBP < 90)		Unadjusted OR (95% CI), P-Value	Adjusted^a^ OR (95% CI), P-Value
	Uncontrolled (n = 504) n (%)	Controlled (n = 285) n (%)		
**Sex**			P = 0.327	
Male	171 (33.93)	87 (30.53)	0.86 (0.63, 1.17)	
Female	333 (66.07)	198 (69.47)	Ref	
**Age**			P = 0.574	
18–39	37 (7.34)	27 (9.47)	Ref	
40–59	229 (45.44)	128 (44.91)	0.77 (0.45, 1.32)	
≥ 60	238 (47.22)	130 (45.61)	0.75 (0.44, 1.28)	
**Educational level**			P = 0.705	
Illiterate	131 (25.99)	80 (28.07)	Ref	
Primary	78 (15.48)	46 (16.14)	0.97 (0.61, 1.53)	
Secondary	139 (27.58)	82 (28.77)	0.97 (0.65, 1.43)	
High school certificate and above	156 (30.95)	77 (27.02)	0.81 (0.55, 1.19)	
**Per Capita Income**			P = 0.927	
Median and Below (< Rs 46,089)	473 (93.85)	267 (93.68)	Ref	
Above Median (> Rs 46,095)	31 (6.15)	18 (6.32)	1.03 (0.56, 1.87)	
**Waist Circumference**			P = 0.066	
Low (< 80 cm for females and < 94 cm for males)	110 (21.83)	82 (28.77)	Ref	
Moderate (80-87.9 cm for females and 94-101.9 cm for males)	112 (22.22)	51 (17.89)	0.61 (0.39, 0.95)	
High (≥ 88 cm for females and ≥ 102 cm for males)	282 (55.95)	152 (53.33)	0.72 (0.51, 1.02)	
**BMI**			**P < 0.001**	**P < 0.001**
< 18.5	6 (1.19)	21 (7.37)	Ref	Ref
18.5 < 23	95 (18.85)	61 (21.40)	0.18 (0.07, 0.48)	0.18 (0.07, 0.47)
23 < 24.9	85 (16.87)	42 (14.74)	0.14 (0.05, 0.38)	0.13 (0.05, 0.34)
≥ 25.0	318 (63.10)	161 (56.49)	0.14 (0.06, 0.37)	0.13 (0.05, 0.34)
**Tobacco consumption**			P = 0.083	
Yes	67 (13.29)	26 (9.12)	0.66 (0.41, 1.06)	
No	437 (86.71)	259 (90.88)	Ref	
**Alcohol consumption**			P = 0.538	
Yes	51 (10.12)	25 (8.77)	0.85 (0.52, 1.41)	
No	453 (89.88)	260 (91.23)	Ref	
**Sedentary lifestyle**			P = 0.193	
Yes	149 (29.56)	97 (34.04)	1.23 (0.90, 1.68)	
No	355 (70.44)	188 (65.96)	Ref	
**Family history of HTN**			P = 0.160	
Yes	60 (11.9)	44 (15.44)	1.35 (0.89, 2.06)	
No	444 (88.1)	241 (84.56)	Ref	
**DM comorbidity**			P = 0.464	
Yes	157 (31.15)	96 (33.68)	1.12 (0.82, 1.53)	
No	347 (68.85)	189 (66.32)	Ref	
**Medication Adherence**			**P = 0.007**	**P = 0.014**
No	232 (46.03)	103 (36.14)	Ref	Ref
Yes	272 (53.97)	182 (63.86)	1.51 (1.12, 2.03)	1.48 (1.08, 2.02)
**Alternative Medicine (AYUSH)**			P = 0.139	
No	488 (96.83)	281 (98.6)	Ref	
Yes	16 (3.17)	4 (1.4)	0.43 (0.14, 1.31)	

Model Goodness of Fit, P = 0.6771

^**a**^Adjusted for education level, BMI and medication adherence

A linear regression was performed, adjusting the systolic and diastolic blood pressure values with sociodemographic and lifestyle variables (Table 5). Among the participants, males (adjusted B = 8.14, 95% CI: 7.25, 9.03), those aged 60 and above (adjusted B = 17.73, 95% CI: 16.56, 18.90), high waist circumference (adjusted B = 6.27, 95% CI: 5.00, 7.55), BMI ≥ 25.0 (adjusted B = 10.88, 95% CI: 9.44, 12.31), alcohol consumers (adjusted B = 2.41, 95% CI: 1.07, 3.75) and those with DM comorbidity (adjusted B = 3.02, 95% CI: 1.52, 4.52) had a significantly higher systolic BP as compared to their respective counterparts. Similarly, males (adjusted B = 5.00, 95% CI: 4.38, 5.61), those aged 60 and above (adjusted B = 4.86, 95% CI: 4.05, 5.67), high waist circumference (adjusted B = 3.91, 95% CI: 3.04, 4.79), BMI ≥ 25.0 (adjusted B = 9.09, 95% CI: 8.10, 10.08), tobacco users (adjusted B = 1.41, 95% CI: 0.54, 2.28), alcohol consumers (adjusted B = 2.30, 95% CI: 1.38, 3.23) and those with a family history of HTN (adjusted B = 1.72, 95% CI: 1.00, 2.44) had a significantly higher diastolic BP as compared to their respective counterparts.

**Table 5:** Distribution of factors associated with systolic and diastolic blood pressure values (N = 8850)

Variables	Systolic blood pressure		Diastolic blood pressure	
	Unadjusted B (95% CI), P-Value	Adjusted^a^ B (95% CI), P-Value	Unadjusted B (95% CI), P-Value	Adjusted^b^ B (95% CI), P-Value
**Sex**	**P < 0.001**	**P < 0.001**	**P < 0.001**	**P < 0.001**
Male	6.60 (5.80, 7.41)	8.14 (7.25, 9.03)	4.41 (3.87, 4.94)	5.00 (4.38, 5.61)
Female	Ref	Ref	Ref	Ref
**Age (years)**	**P < 0.001**	**P < 0.001**	**P < 0.001**	**P < 0.001**
18–39	Ref	Ref	Ref	Ref
40–59	12.17 (11.33, 13.01)	8.54 (7.70, 9.38)	7.11 (6.52, 7.69)	4.67 (4.09, 5.25)
≥ 60	20.84 (19.70, 21.99)	17.73 (16.56, 18.90)	6.20 (5.41, 7.00)	4.86 (4.05, 5.67)
**Educational level**	**P = 0.0022**	**P = 0.0467**	P = 0.2544	P = 0.2131
Illiterate	Ref	Ref	Ref	Ref
Primary	-0.53 (-1.83, 0.77)	-0.36 (-1.48, 0.76)	-0.09 (-0.95, 0.76)	-0.31 (-1.08, 0.47)
Secondary	-2.07 (-3.20, -0.95)	-1.33 (-2.31, -0.36)	-0.37 (-1.11, 0.38)	-0.68 (-1.35, -0.003)
High school certificate and above	-1.05 (-2.18, 0.09)	-0.59 (-1.57, 0.40)	0.33 (-0.42, 1.08)	-0.19 (-0.87, 0.49)
**Per Capita Income**	P = 0.471		P = 0.779	
Median and Below (< Rs 46,089)	Ref		Ref	
Above Median (> Rs 46,095)	-0.59 (-2.20, 1.02)		-0.15 (-1.22, 0.91)	
**Waist Circumference**	**P < 0.001**	**P < 0.001**	**P < 0.001**	**P < 0.001**
Low (< 80 cm for females and < 94 cm for males)	Ref	Ref	Ref	Ref
Moderate (80-87.9 cm for females and 94-101.9 cm for males)	7.52 (6.50, 8.55)	3.79 (2.69, 4.90)	4.76 (4.08, 5.44)	2.22 (1.46, 2.98)
High (≥ 88 cm for females and ≥ 102 cm for males)	10.51 (9.58, 11.44)	6.27 (5.00, 7.55)	6.60 (5.98, 7.22)	3.91 (3.04, 4.79)
**BMI**	**P < 0.001**	**P < 0.001**	**P < 0.001**	**P < 0.001**
< 18.5	Ref	Ref	Ref	Ref
18.5 < 23	7.45 (6.13, 8.77)	6.57 (5.38, 7.76)	5.42 (4.56, 6.28)	4.98 (4.16, 5.80)
23 < 24.9	13.00 (11.53, 14.48)	9.81 (8.42, 11.20)	9.46 (8.49, 10.42)	7.75 (6.79, 8.71)
≥ 25.0	16.20 (14.91, 17.49)	10.88 (9.44, 12.31)	12.00 (11.16, 12.84)	9.09 (8.10, 10.08)
**Tobacco consumption**	**P < 0.001**	P = 0.157	**P < 0.001**	**P = 0.002**
Yes	6.90 (5.77, 8.04)	0.91 (-0.35, 2.17)	4.96 (4.21, 5.71)	1.41 (0.54, 2.28)
No	Ref	Ref	Ref	Ref
**Alcohol consumption**	**P < 0.001**	**P < 0.001**	**P < 0.001**	**P < 0.001**
Yes	7.26 (6.05, 8.48)	2.41 (1.07, 3.75)	5.75 (4.95, 6.55)	2.30 (1.38, 3.23)
No	Ref	Ref	Ref	Ref
**Sedentary lifestyle**	**P < 0.001**	P = 0.783	P = 0.200	
Yes	1.83 (0.90, 2.77)	-0.11 (-0.93, 0.70)	-0.40 (-1.02, 0.21)	
No	Ref	Ref	Ref	
**Family history of HTN**	P = 0.704		**P < 0.001**	**P < 0.001**
Yes	0.23 (-0.97, 1.43)		1.87 (1.08, 2.67)	1.72 (1.00, 2.44)
No	Ref		Ref	Ref
**DM comorbidity**	**P < 0.001**	**P < 0.001**	**P < 0.001**	P = 0.346
Yes	14.08 (12.46, 15.70)	3.02 (1.52, 4.52)	4.38 (3.30, 5.47)	-0.50 (-1.53, 0.54)
No	Ref	Ref	Ref	Ref

^**a**^Adjusted for sex, age, education level, waist circumference, BMI, tobacco consumption, alcohol consumption, sedentary lifestyle and DM comorbidity. Adjusted R-squared = 0.2608

^**b**^Adjusted for sex, age, education level, waist circumference, BMI, tobacco consumption, alcohol consumption, family history of HTN and DM comorbidity. Adjusted R-squared = 0.1968

## Discussion

The present study investigated the prevalence and risk factors of hypertension among adults aged 18 years and over in an urban slum area of Delhi, India. The hypertension care cascade in the present study indicates that nearly 29% of the participants were hypertensive of which 61.77% were newly diagnosed cases, 81.4% of previously diagnosed cases were initiated on antihypertensive medication of which 57.54% were adherent to their medications, while 36.12% attained controlled blood pressure levels. The prevalence of hypertension in this study (~ 29%) is higher than the burden observed in a study conducted in an urban slum of Mumbai (23.6%) [39] and also the India country level prevalence among young and middle-aged population (NFHS-5) (22.8%) [15]. However, the overall prevalence is much lower compared to the burden among older adult and elderly population estimates from nationally representative survey data from India (LASI) (36%) [17]. The burden of hypertension in the present study was also nearly similar to that observed in populated aged ≥ 35 years from urban slum areas in Bangladesh (28.3%) [29], higher compared to urban slums in Brazil (12.3%) [28] but lower than urban slums in Kolkata (42%) [30]. Our study found the prevalence of hypertension to be higher in males (32.21%) than females (26.25%). These proportions are higher than the national estimates in the general populated aged 15–49 years as per NFHS 5 (2019–2021) (males, 24% and females, 21%) [40].

Advancing age and alcohol consumption were found to be positively associated with the odds of having hypertension, a finding corroborated from previous evidence [5]. The prevalence of hypertension for the adults aged 45 years and older was found to be 48.51% in our sample, higher than that found from LASI Wave 1 (2017–2018) (45.9%) [41]. Furthermore, high waist circumference was also identified as a significant predictor of hypertension corroborating the evidence from previous studies [42, 43]. However, diabetes status was not an independent predictor of hypertension in this study compared to observations from LASI probably because of the older population sample with greater risk factors in the latter [17].

Nearly one in five participants had undiagnosed hypertension in the present study, a finding which indicates the need for intensification of screening, both opportunistic and community-based in resource limited settings. Furthermore, male gender and increasing age were associated with undiagnosed hypertension in this study, findings consistent with a single site study conducted in Puducherry, a town in Southern India [44]. Moreover, in the present study, a high burden of undetected hypertension was independently associated with behavioural/physiological risk factors (moderate-high waist circumference, increasing BMI and alcohol consumption) suggesting an early social transition of cardiovascular disease in the urban poor and the need for integrating and prioritizing lifestyle interventions within primary and secondary prevention strategies for hypertension care and control in India.

In this study we observed nearly one in five previously diagnosed hypertensive cases were not initiated or not taking any anti-hypertensive medications signifying major deficiencies in the continuum of care as these patients possibly did not report back to their health system, a finding also observed in a study in slums in Bangladesh [29]. Furthermore, a majority of the patients on treatment reported medication nonadherence, a factor which was independently associated with poorly controlled blood pressure levels. Adverse social determinants of health prevalent in urban slum dwellers including poor-socioeconomic status, illiteracy, unemployment, lack of awareness, and out of pocket medication expenses are possible reasons for non-adherence [45, 46].

Current tobacco users in previously diagnosed hypertension cases in this study was 113 (11.66%), a finding lower than that observed in cross-sectional studies from slums in Bangladesh (27.33%) [29], Kolkata (44.35%) [30], Kenya (32.3%) [47] and Egypt (43.65%) [48]. Among those who smoked tobacco, 41.6% women and 35.51% men were hypertensive in our study, comparatively higher than the findings from NFHS-4 (2015–2016), where 15.3% women and 22.4% men who smoked tobacco were found hypertensive [49]. Promoting healthcare readiness to provide regular tobacco cessation services in the same health facility to patients where they are undergoing hypertension management is crucial since tobacco users apart from increased risk of cancers also contribute independently to an elevated risk of cardiovascular disease, similar to hypertension, and both may synergistically interact to accentuate this potential risk in the patients. The overall control of hypertension was suboptimal even among those reporting taking antihypertensive treatment (36.1%) suggestive of the presence of clinical inertia or failure of intensification of therapy by physicians despite persistently poorly controlled blood pressure levels. This proportion of patients with controlled blood pressure is lower than that reported in studies from Chennai (45.9%) [50], while greater than Kerala (32.1%) [51] and Kolkata (26%) [30] but indicative of a nationally prevalent problem. Another study conducted in urban slums of Kolkata among patients with hypertension reported that patients adherent to antihypertensive medications were 1.71 times more likely to achieve adequate blood pressure control compared to non-adherent patients, a finding similar to the present study [52].

The study has several strengths including a large sample size ensuring validity of the data, while representativeness was high when considering the problem in urban slum settings. Furthermore, all components of a disease care cascade including medication adherence, an information which is not usually collected in nationally representative secondary surveys were incorporated in this study. However, there are certain limitations to this study. Firstly, the findings of the present study should not be generalized to people living in improved non-slum conditions although we were able to compare the hypertension burden and care cascade outcomes in the present study with estimates derived from multiple large nationally representative datasets. Second, being cross-sectional in nature, the study could not establish causality due to lack of temporal evidence between hypertension and its determinants. Third, some selection bias is possible as those adults who were not available in the household during the survey period were omitted. Similarly. Recall bias could have contributed to overestimation of previously diagnosed cases of hypertension, while social desirability bias may have contributed to inflated measures of antihypertensive medication adherence. Fourth, we did not ascertain adherence to prescribed healthy diet such as salt restriction and consumption of fresh fruits and vegetables in the participants which can influence blood pressure control [53]. Fourth, as samples were selected through clusters, the possibility of clustering of risk factors in the regression analysis despite checking for multicollinearity cannot be completely ruled out. Fifth, our blood pressure measurements could have some variation due to the white coat effect that may cause a slight elevation in the readings although this phenomenon is unavoidable in large epidemiological surveys [54].

Our study has some important implications for enhancing the hypertension care and control cascade in slum settings in India and similar low-resource settings in LMICs. First, despite the mandate for annual community-based population screening for hypertension by frontline health workers (FHWs), a large proportion of existing patients with hypertension in slum residing communities remain undiagnosed signifying gaps and inefficient screening [31]. Refresher training and sensitization of FHWs is crucial since our findings indicate that conducting rapid community surveys for screening hypertension is feasible in such settings. Second, information, education, communication (IEC) campaigns to increase public demand for getting screened for hypertension and adopting healthy lifestyles as preventative strategies warrants high prioritization. Finally, ensuring adherence support for existing patients on antihypertensive medication from nursing and pharmacy staff at primary health facilities, and sensitization and training of doctors to avoid clinical inertia in patients with uncontrolled hypertension and ensure adequacy of drug treatment is an ethical imperative for medical practitioners in slum associated low-resource settings.

In conclusion, hypertension care cascade in urban slum-resettlement colony setting revealed a high burden of undiagnosed hypertension, low rates of medication adherence, and poor blood pressure control. Strengthening community screening and primary care continuum of care is necessary to improve the hypertension care cascade from early diagnosis to effective management with optimal health outcomes to reduce patient complications and increase longevity. Future research should ascertain the individual, community, and health-system factors responsible for missed or delayed screening, diagnosis, and initiation of antihypertensive treatment in urban slum areas.

## Data Availability

The dataset used and analysed during the current study are available from the corresponding author on reasonable request.
